# Thrombomodulin Regulation of Mitogen-Activated Protein Kinases

**DOI:** 10.3390/ijms20081851

**Published:** 2019-04-15

**Authors:** Hemant Giri, Xiaofeng Cai, Sumith R. Panicker, Indranil Biswas, Alireza R. Rezaie

**Affiliations:** 1 Cardiovascular Biology Research Program, Oklahoma Medical Research Foundation, Oklahoma City, OK 73104, USA; hemant-giri@omrf.org (H.G.); xiaofeng-cai@omrf.org (X.C.); sumith-panicker@omrf.org (S.R.P.); indranil-biswas@omrf.org (I.B.); 2 Department of Biochemistry and Molecular Biology, University of Oklahoma Health Sciences Center, Oklahoma City, OK 73104, USA

**Keywords:** thrombomodulin, mitogen-activated protein kinases (MAPKs), inflammation

## Abstract

The multifaceted role of mitogen-activated protein kinases (MAPKs) in modulating signal transduction pathways in inflammatory conditions such as infection, cardiovascular disease, and cancer has been well established. Recently, coagulation factors have also emerged as key players in regulating intracellular signaling pathways during inflammation. Among coagulation factors, thrombomodulin, as a high affinity receptor for thrombin on vascular endothelial cells, has been discovered to be a potent anti-inflammatory and anti-tumorigenic signaling molecule. The protective signaling function of thrombomodulin is separate from its well-recognized role in the clotting cascade, which is to function as an anti-coagulant receptor in order to switch the specificity of thrombin from a procoagulant to an anti-coagulant protease. The underlying protective signaling mechanism of thrombomodulin remains largely unknown, though a few published reports link the receptor to the regulation of MAPKs under different (patho)physiological conditions. The goal of this review is to summarize what is known about the regulatory relationship between thrombomodulin and MAPKs.

## 1. Introduction

### 1.1. Mitogen-Activated Protein Kinases (MAPKs)

Mitogen-activated protein kinases (MAPKs) are protein serine/threonine (Ser/Thr) kinases involved in regulation of various physiological processes including proliferation, differentiation, migration and apoptosis [1]. They coordinate cellular responses to environmental cues (i.e., hormones) by phosphorylating intracellular signaling molecules on distinct Ser/Thr residues, thereby initiating and integrating a number of phosphorylation cascades which are involved in the regulation of different signal transduction pathway genes [2]. The kinase function of MAPKs is tightly regulated by phosphatases, protein–protein interactions and proteases and their misregulation can cause pathological conditions including chronic inflammation, diabetes, neurodegenerative disease, cardiovascular disease and cancer [3]. The most studied MAPKs are classified into three types; extracellular signal-regulated kinases (ERKs), c-Jun N-terminal kinases (JNKs), and 38 kDa mitogen-activated protein kinase (p38 MAPK) [1]. The activation of ERK1/2 is thought to be primarily induced by growth factors that are mainly observed during cell growth and differentiation [3,4]. However, p38 and JNK MAPKs are mainly activated during immune cell responses under conditions of oxidative stress and inflammation [3,4,5]. Hence, the last two MAPKs are classified as stress-activated protein kinases (SAPKs), and inhibitors of SAPKs are widely used in inflammatory disorders and cancer to control the excessive SAPKs signaling [6,7].

### 1.2. Thrombomodulin

Thrombomodulin (TM) is a transmembrane glycoprotein with 575 amino acids which was first discovered by Esmon and Owen in 1981 [8]. It was originally discovered on endothelial cells and later found to express at various abundance on other cell types, such as trophoblasts of the placenta [9], platelets [10], megakaryocytes [11], leukocytes [12,13], keratinocytes [14], and astrocytes [15]. TM plays an important modulatory role in coagulation biology by functioning as a cofactor for activation of protein C to activated protein C (APC) by thrombin on vascular endothelial cells [8]. APC has anti-coagulant, anti-inflammatory and anti-apoptotic properties, thus providing a thromboresistant and cytoprotective phenotype to injured vessels during which coagulation and inflammatory pathways are upregulated [16]. APC mediates its anti-coagulant effect by degrading activated cofactors V and VIII by limited proteolysis, while anti-inflammatory and cytoprotective effects are mediated through APC binding to endothelial protein C receptor (EPCR) and activating protease-activated receptor 1 (PAR1) on endothelial cells [17]. Loss of TM mediated APC generation causes thrombosis in TM knock-out mice [18] as well as in mice containing mutant TM [19]. Genetic supplementation of APC in TM knock-out mice has been demonstrated to reduce coagulation and fibrinolytic defects, however, it has not attenuated increased lung vascular permeability and plasma IL-6 levels, suggesting that TM has an APC-independent anti-inflammatory function [20]. Anti-coagulation and anti-inflammatory properties of TM have led to the approval of a soluble form of TM lacking its cytosolic and transmembrane domains as a drug for the treatment of disseminated intravascular coagulation (DIC) and sepsis in Japan [21]. The human soluble recombinant TM (rTM), commercial name: ART-123, was tested in clinical trials performed at various countries. It has been found that it is effective in reducing inflammation, levels of coagulopathy markers such as D-dimer, TAT complexes and mortality among patients with sepsis [22].

As indicated above, as an anti-coagulant receptor for thrombin, the mechanism by which TM functions in the coagulation cascade is relatively well understood, however, the intracellular signaling mechanism of TM that regulates inflammation remains largely unknown and is under intensive investigation by several groups [23,24,25,26]. Here, we briefly review the current knowledge that links thrombomodulin to MAPK signaling during inflammation.

## 2. Structure of TM and Significance of Its Various Domains in the Regulation of MAPK Signaling

The structure of TM is composed of several distinct domains including an N-terminal lectin-like domain (LD) (1–154 residue), a hydrophobic region (156–222 residue), six epidermal growth factor (EGF)-like domains (223–462 residue), a membrane proximal Ser/Thr-rich region containing N– & O–glycosylation sites, a transmembrane domain (463–497 residue), and a cytoplasmic tail (522–557 residue) [24].

### 2.1. Lectin-Like Domain

It has been well-established that the lectin-like domain of TM exhibits a potent anti-inflammatory function that inhibits NF–κB activation, expression of cell adhesion molecules (CAMs) and the adhesion of neutrophils to the endothelium [23]. Moreover, it has been demonstrated that the lectin-like domain interacts with specific carbohydrate moieties present on inflammatory ligands such as high mobility group box 1 (HMGB1) and lipopolysaccharides (LPS), thereby sequestering them on the surface of inflamed cells and attenuating their interaction with pro-inflammatory receptors [23]. It has been discovered that binding of lectin-like domain to HMGB1 also leads to its cleavage by thrombin and that TM significantly increases the cleavage and inactivation of HMGB1 by thrombin [27]. This results in reduced availability of HMGB1 for interaction with its receptors including toll-like receptors (TLR) 2 and 4 and receptor for advanced glycation end products (RAGE) [28]. HMGB1 signaling is known to elicit potent pro-inflammatory signaling responses through MAPK signaling and its TM-mediated sequestration and/or cleavage blocks this process, suggesting that TM is involved in inhibiting MAPK signaling through this mechanism [27]. By a similar mechanism, lectin-like domain binds its ligand Lewis Y antigen on LPS and neutralizes the inflammatory effects of LPS and its associated MAPK signaling [29]. In this study, lectin-like domain has been found to decrease the LPS-induced production of tumor necrosis factor-α (TNFα), NF–κB signaling and phosphorylation of ERK1/2 and p38 MAPKs in RAW 264.7 cells [29]. A similar effect was reported in in vivo, where intravenous injection of lectin-like domain attenuated the serum levels of TNFα in mice challenged with LPS or *Klebsiella pneumoniae* [29].

In keratocytes, the lectin-like domain peptide inhibits LPS-induced expression of ICAM and p65 by suppressing the activation of NF-κB and MAPKs (p38 and JNK) pathways [30]. P38 and JNK seem to be the main targets of TM since the LPS-induced phosphorylation of ERK was unaltered upon treatment with lectin-like domain peptide [30]. Deletion of the lectin-like domain in mice has no phenotype [31]. However, challenging these mice with LPS results in elevated cytokine production, increased activation of ERK1/2, and higher mortality as compared to wild-type mice treated with a similar dose of LPS [31]. Interestingly, the activation of protein C was found to be normal in these mice suggesting that lectin-like domain of TM itself has a potent anti-inflammatory function independent of APC [31]. The possible direct or indirect roles of different TM domains in regulating MAPK signaling pathways under different pathophysiological conditions are presented in Table 1.

### 2.2. EGF-Like Domains

A recombinant TM peptide composed of six EGF-like domains (rTME1-6) induces proliferation, and accelerates DNA synthesis and glucose uptake in the Swiss 3T3 fibroblast cell line, indicating that this mitogenic activity could be due to the binding of rTME1-6 to a different site from the EGF receptor on the cell surface [46]. Similarly, a recombinant protein composed of EGF–like domains 1-6 plus the Ser/Thr-rich domain of TM (rTMD23) induces DNA synthesis in cultured human umbilical vein endothelial cells (HUVECs) [39]. Additionally, rTMD23 stimulates chemotactic motility and capillary like tube formation in HUVECs through activation of ERK1/2 and phosphoinositide 3-kinase (PI3K) signaling pathways [39], further confirming the mitogenic and angiogenic properties of TM via EGF domains and/or Ser/Thr-rich domain in in vitro cell culture system. Apart from its angiogenic effects, rTMD23 has been found to reduce thrombin-induced PAR1 internalization, elevation in the cytosolic calcium concentration, expression of cell adhesion molecules (ICAM-1 and VCAM-1) and chemokines (MCP-1 and TNFα) in HUVECs, human aortic endothelial cells (HAECs) and mouse macrophage cell line RAW264.7 [40]. Furthermore, rTMD23 decreases thrombin-mediated permeability in HUVECs and HAECs [40]. It has been demonstrated that rTMD23 also prevents atherosclerosis in apolipoprotein E–deficient mice by binding to thrombin and inhibiting thrombin-induced endothelial cell dysfunction [40]. Pretreatment or posttreatment of rTMD23 has dramatically decreased inflammatory responses and increased the survival rate in a mouse model of LPS-induced sepsis [41]. It has been found that rTMD23 suppresses LPS-induced ERK1/2 activation and inflammatory responses by targeting CD14 in mouse peritoneal macrophages [41], thus suggesting a crosstalk between TMD2&3 and MAPKs.

### 2.3. C-Terminal Domain

TM cytoplasmic tail or the C-terminal domain of TM does not have intrinsic enzymatic activity like receptor tyrosine kinases. The function of C-terminal domain is not well defined in endothelial cells. However, in the monocytic THP-1 cell line, the C-terminal domain of TM has been found to be essential for inhibition of IL-6-enhanced chemotaxis and actin assembly [45]. It induces the interaction of actin with cytoplasmic adaptor proteins, culminating in activation of ERK and JNK signaling pathways [45]. In addition, in epithelial cells such as A431, the C-terminal domain of TM maintains the cellular morphology and promotes collective cell migration by direct interaction with ezrin in cell–cell contacts, which connects the TM to actin filaments [47]. Furthermore, TNFα-induced activation of p38 MAPK leads to phosphorylation of ezrin and downregulation of TM, although p38 does not directly phosphorylate ezrin [48]. This results in disassociation of interaction among TM C-terminal domain, ezrin and actin filaments, thereby causing loss of barrier function [48]. In mice, deletion of the C-terminal domain does not affect embryonic development, inflammation, coagulation, and skin wound healing [49]. Thus, more studies are needed to understand the specific physiological role of the C-terminal domain of TM.

## 3. Thrombomodulin Crosstalk with Other Receptors Modulating MAPK Signaling

Binding of thrombin to TM on endothelial cells cleaves EPCR-bound protein C to generate APC [50]. APC exerts potent anti-coagulant, anti-inflammatory and cytoprotective effects as discussed earlier [16]. APC attenuates LPS and IL-1-induced p38 MAPK activation through EPCR/PAR1/S1P1-dependent mechanisms in the porcine model of endotoxic shock as well as in HUVECs [42]. Similarly, APC reduces TNFα-induced phosphorylation of p38 MAPK and JNK in rheumatoid synovial fibroblasts [43]. Apart from its inhibitory effect on MAPKs, APC activates ERK1/2 during endothelial cell proliferation and angiogenesis, as confirmed in in vitro angiogenesis (HUVECs Matrigel assay) and in vivo mouse corneal angiogenesis model systems [51]. There is a report indicating that APC activates ERK1/2 pathway to increase the expression of early growth response factor−1 (EGR-1), a negative regulator of TRAIL expression, to subsequently inhibit TNFα-induced apoptosis in endothelial cells through PAR1/S1P1-dependent but EPCR-independent mechanisms [52]. Thus, the activation of protein C by thrombin, facilitated by TM, is one of the key pathways through which TM can regulate MAPK signaling pathways.

Recently, RAGE has been found to regulate the generation of APC along with EPCR and TM [53]. Reduced protein C activation was observed in cultures of RAGE knock-out murine aortic endothelial cells [53]. These cells were shown to have decreased mRNA expression levels of both EPCR and TM [53], thus explaining possible reasons for low APC generation on these cells. In addition to APC generation, TM can directly suppress thrombin-induced endothelial cell activation triggered by the cleavage of PAR1 [54]. Thrombin binding to PAR1 leads to proliferation of endothelial cells by phosphorylating cytoplasmic ERK1/2 to induce its translocation to the nucleus, where phosphorylated ERK (pERK) further coordinately activates other nuclear proteins and it may also return back to the cytoplasm to repeat this cycle [55,56]. However, interaction with TM attenuates all of these effects mediated by thrombin through increasing the nuclear retention of pERK [55], which suggests that in addition to its regulatory role in blood coagulation, TM acts as a thrombin receptor to modulate the duration of pERK nuclear retention and cell proliferation in response to inflammatory stimuli.

It has been recently demonstrated that interaction of EGF5 domain of TM with another receptor, G-protein coupled receptor 15 (GPR15), induces angiogenesis and cell survival through activation of ERK and BCL-2 in HUVECs and in murine aortic endothelial cells [57], suggesting that GPR15 plays an important role in mediating cytoprotective and angiogenic functions of TM. In addition, rTMD2&3 interacts with fibroblast growth factor receptor (FGFR1) to induce angiogenesis independent of the APC pathway, as shown in HUVECs tube formation assay and the corneal angiogenesis model in BALB/c mice [58]. In smooth muscle cells, TM activates ERK pathway through the EGFR axis, thereby downregulating growth arrest-specific gene 6 (Gas6) [59]. Gas6 is a key molecule involved in stimulation of cell proliferation and its downregulation has been shown to lead to vascular calcification in rats [59]. Taken together, these studies provide support for a crosstalk between TM and other receptors in regulating MAPK signaling which may have physiological significance.

## 4. Thrombomodulin and MAPK Signaling in Leukocytes

Decreased expression of TM on monocytes was reported in patients with DIC [60], coronary artery bypass graft surgery [61] and during osteoclastogenesis (inflammatory bone loss) [37]. These pathological conditions are associated with increased activation of MAPKs including p38, JNK and ERK1/2 [62,63]. Osteoclastogenesis was also observed in macrophages derived from myeloid TM deleted mice, suggesting an anti-inflammatory role for TM in circulating cells [37]. Use of recombinant TM protein improves the survival of DIC patients [64] and attenuates the inflammatory bone loss in collagen antibody-induced arthritis and ovariectomy-induced mice models [37]. Apart from these direct effects, TM mediated generation of APC has been shown to reduce LPS-induced secretion of TNFα, IL-1β, IL-6, and IL-8 from human monocytes and THP-1 cell line [65]. This is also associated with a reduction in LPS-induced apoptosis and adhesion of human monocytes to the endothelium [65]. A similar observation has been reported in bronchoalveolar lavage (BAL) obtained from rats treated with LPS and APC [44]. In addition to decreased cytokine levels, reduced numbers of leukocytes and neutrophils were observed in BAL fluid [44]. APC mediates these anti-inflammatory and anti-apoptotic effects by inhibiting the activation of p38, ERK1/2, JNK [44], and NF-κB [66] signaling pathways.

In addition to its anti-inflammatory function, TM can have an effect on regulation of monocyte differentiation and this effect requires activation of protein kinase C-δ (PKCδ) and ERK1/2 pathways [67]. Interaction of PKCδ with TM in the monocytic THP-1 cells leads to inhibition of the cell cycle and subsequent differentiation through ERK1/2 mediated cytoskeletal remodeling [67]. Moreover, monocytic TM is reported to bind Lewis-Y (LeY) on the inflamed endothelium, thereby triggering an increase in activation of p38 MAPK signaling [68]. This facilitates firm binding of THP-1 to ICAM-1 on the endothelium by activating β2 integrins on THP-1 cells [68]. However, this process seems to operate under localized inflammatory conditions since systemic inflammatory molecules like LPS decreases the TM expression on THP-1 cells [69]

## 5. Thrombomodulin and MAPK Signaling in Platelets

Platelets amplify inflammatory responses by secretion of a large number of pro-coagulant and pro-inflammatory cytokines such as adenosine diphosphate (ADP), Thromboxane A_2_ (TxA_2_), chemokines CXCL4 (platelet factor 4), CCL5 (RANTES), platelet-derived growth factor (PDGF), CXCL7 (Neutrophil-activating peptide-2, NAP-2) [70] and HMGB1 [71]. Thrombocytopenia is the first response to any infection which indicates that platelets are engaged in the resolution of infection [72]. During this process, platelets directly interact with neutrophils, monocytes, endothelial cells and pathogens to amplify the inflammatory response [72]. MAPKs and NF-κB signaling pathways play important roles in activation of platelets during thrombosis and inflammation [73,74]. The inhibitory function of soluble TM on platelet activation by thrombin has been reported [75]. Addition of soluble TM inhibited thrombin-induced aggregation and serotonin release in human platelets [75]. Similarly, addition of recombinant TM to washed platelets inhibited histone induced aggregation [32]. In mice, intravenous injection of histones leads to thrombocytopenia and mortality, which is reversed by pretreatment with recombinant TM [32]. Histones are known to cause platelet activation and increased platelet–leukocyte interaction [76]. These effects of histones are mediated through TLR2/4 receptors which activate ERK and p38 MAPKs and NF-κB pathways [76]. This suggests that TM could interact directly with histones to inhibit the initiation of MAPK signaling in platelets [32].

Platelets play a major role in the pathogenesis of deep vein thrombosis (DVT) [77]. This process involves binding of leukocytes on the venous endothelium, which is followed by binding of platelets and formation of platelet–leukocytes aggregates [77]. Activated platelets interact with neutrophils, leading to formation of neutrophil extracellular traps (NETs) [77]. A recent study demonstrated that activated platelets secrete HMGB1, which is associated with NETs formation by neutrophils [71]. Platelet-derived HMGB1 enhances neutrophil recruitment, their activation and NETs formation in a murine model of deep vein thrombosis [71]. Interestingly, addition of recombinant TM to the co-culture of neutrophils and platelets inhibits the NETs formation in response to LPS [33]. Similarly, intravenous injection of recombinant TM inhibits venous thrombosis in rats [34]. In humans, use of soluble TM (ART-123) reduces venous thrombosis in patients who have undergone total hip replacement [35]. This indicates that TM inhibits NF-κB, MAPKs and HMGB1 signaling, thereby reducing platelet activity under pathological conditions.

## 6. Thrombomodulin Inhibits Proliferation and Tumor Invasion by Inhibiting MAPKs

Signaling mechanisms promoting tumor progression and invasion involve several MAP kinases including p38, JNK and ERK1/2 [78]. Numerous studies have shown a protective effect for TM in tumorigenesis and metastasis and a decreased TM expression has been reported to exhibit loss of differentiation and enhanced metastatic property for tumors [79,80]. However, the signaling mechanism by which TM mediates its anti-tumorigenesis activity remains largely unknown. In lung cancer cells, TM was found to reduce tumorigenic and metastatic potential by up-regulation of E-cadherin and downregulation of N-cadherin [81] and in patients with resected hepatocellular carcinoma, TM was proposed to prevent intrahepatic metastasis [82]. Recombinant lectin-like domain of TM was found to inhibit tumor angiogenesis in a murine Matrigel implantation assay and in a rat corneal micropocket assay [38]. In patients with colorectal cancer, pancreatic cancer and various other cancers in the terminal stage, the plasma levels of soluble TM was found be enhanced [83]. Thrombin binding to PAR1 leads to tumor growth and hypertrophy through activation of p38, JNK, ERK5, ERK6 and increased transcription of c-Jun [84,85]. Recently, use of recombinant TM has been shown to reduce PAR1-induced tumor growth in pancreatic cancer [36]. This anti-proliferative effect of TM has also been observed in endothelial cells, where TM increases thrombin-induced ERK nuclear retention and thereby inhibits endothelial cell proliferation [55,86]. Interestingly, it was previously reported that a specific domain of TM can inhibit monocyte cytoskeletal rearrangement and migration by inhibiting ERK1/2 and JNK/SAPK activation [45]. In another study TM was found to suppress polymorphonuclear cell adhesion to endothelial cells and prevent NF-κB and ERK1/2 activation [31]. Therefore, it will be of great interest to further explore the possibility that TM’s anti-tumor, anti-metastatic and anti-angiogenic effects in various cancers are mainly mediated through negative regulation of MAPK signaling pathways.

## 7. MAPK Represses TM Expression and Activity

Decreased TM expression has been observed under various stress and inflammatory conditions such as atherosclerosis [87], DIC [60], diabetic neuropathy [88], oxidative stress [89], vein graft thrombosis [90] and during tumorigenesis [79,80]. All of these conditions are associated with increased MAPKs signaling, supporting the negative correlation hypothesis between TM and MAPK pathways as discussed above.

### 7.1. Downregulation of TM during Inflammation

Several inflammatory factors are known to downregulate the expression of TM [24]. In endothelial cells, TNFα induces downregulation of TM expression through JNK and p38 pathways [91]. Activation of these kinases leads to formation of a transcription repressor complex composed of activating transcription factor-2 (ATF-2) and histone deacetylase-4 (HDAC4) on the TM promoter [92]. This subsequently leads to histone deacetylation and transcription repression of TM [92]. A similar mechanism of TM repression was reported in HAECs treated with palmitic acid. Furthermore, ATF-2 can inhibit the binding of other transcription factors such as Sp-1, which activates the transcription of TM [92]. Thus, JNK and p38 pathways play important roles in downregulation and elimination of the TM receptor during inflammation and metabolic stress conditions.

Apart from TNFα and fatty acids, extracellular histones are also known to decrease the expression and activity of TM [93]. Histones can be released into blood circulation under various inflammatory conditions such as sepsis [94], DVT [95] and DIC [96]. During inflammation, the release of extracellular traps from neutrophils, mast cells, eosinophils, monocytes and macrophages also plays a critical role in increasing the levels of histone in the blood stream [97]. The addition of calf thymus histones on EA.hy926 cells results in reduced surface expression as well as activity of TM [93].

LPS, major components of the cell wall in Gram negative bacteria, are involved in pathogenesis of sepsis and septic shock [98]. LPS decreases the TM surface expression and its mRNA levels in human peripheral blood monocytes by the NF-κB pathway [69]. A similar effect has been demonstrated in THP-1 cell line treated with LPS under serum starved conditions [69]. Furthermore, an LPS-induced decrease in TM activity and its mRNA level has been observed in the liver sinusoidal endothelial cells isolated from rats [99]. These effects were confirmed in rat model of LPS-induced sepsis, where intraperitoneal injection of LPS leads to decreased immunostaining of TM in rat liver sinusoids [99].

### 7.2. Downregulation/Inactivation of TM during Oxidative Stress

Oxidized low-density lipoprotein (ox-LDL) is a key factor in progression of atherosclerosis [100], which causes endothelial cell apoptosis by the activation of p38 and JNK pathways [101]. Decreased expression of TM has been reported in lesions of atherosclerotic plaques obtained from patients with severe coronary artery disease and ischemic cardiomyopathy (ICMP) [87]. Ox-LDL downregulates the expression of TM by reducing the levels of nuclear transcription factors RARβ, RXRα, Sp1, and Sp3 and their binding to TM promoter in HUVECs [89]. However, the identity of receptor for ox-LDL remained unknown in this previous study.

Other oxidative stress conditions like smoking and diabetes are known to reduce the activity of TM by oxidation of its specific residue, Met388 [102]. Met388 is located on the fifth EGF domain of TM and its oxidation leads to reduced activation of protein C by thrombin in complex with the Met388 oxidized TM [103].

In keratinocytes, UV irradiation-induced oxidative stress and ROS generation leads to downregulation of TM [104]. UV irradiation has been known to activate ERK, JNK and p38 MAPKs through EGF and IL-1α receptors [105]. ERK plays an important role in regulation of TM expression as confirmed by the use of ERK inhibitor (PD98059 or U0126) in these cells [104]. Furthermore, ERK activation increases the nuclear retention of p53, which binds to the TM promoter and causes its repression [104].

### 7.3. Downregulation of TM during Tumorigenesis

During tumorigenesis, activation of MAPK leads to increased expression of snail, a zinc finger transcription factor [106]. Binding of snail to the DNA sequences on the TM promoter significantly suppresses the promoter activity of TM in HaCaT cells, thereby reducing the protein expression of TM [107]. The expression level of TM has been shown to be markedly downregulated in A375 malignant melanoma cells when compared to normal human melanocytes [108]. The transient transfection of A375 cells with a construct coding for human TM significantly reduced the metastatic phenotype of these cells, suggesting that downregulation of TM may play a crucial role in melanocyte transformation and melanoma progression [108]. Taken together, downregulation of TM may be a key mechanism through which MAPKs accelerate inflammation and tumorigenesis.

## 8. Summary

**Figure 1:**
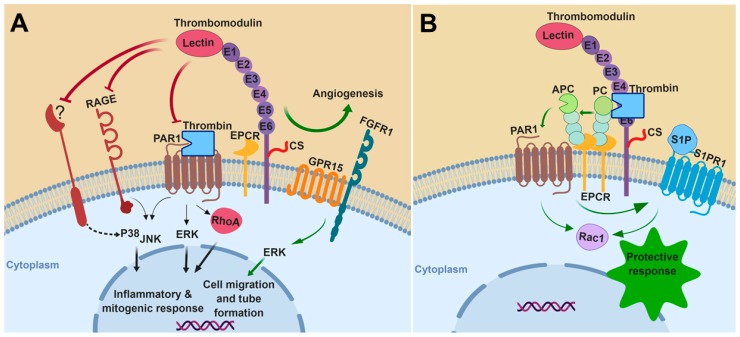
A hypothetical model for the role of thrombomodulin (TM) in mitogen-activated protein kinases (MAPKs) signaling: Thrombomodulin regulates the activation of MAPKs under various pathophysiological conditions. (**A**) Under inflammatory and mitogenic conditions, TM attenuates MAPK pathways by inhibiting the signaling functions of protease-activated receptor 1 (PAR1), receptor for advanced glycation end products (RAGE), and other unknown receptor(s). During angiogenesis, it promotes extracellular signal-regulated kinase (ERK)-mediated vessel growth through activation of protein C (PC) to activated protein C (APC) and crosstalk with other receptors such as G-protein coupled receptor 15 (GPR15) and fibroblast growth factor receptor 1 (FGFR1). (**B**) TM functions as a cofactor for thrombin to mediate the activation of PC to APC, thereby initiating anti-inflammatory and cytoprotective signaling responses through APC-mediated activation of PAR1, inhibition of RhoA and promoting a protective signaling pathway through Rac1. APC trans-activates S1PR1 receptor to enhance the barrier permeability function of endothelial cells. CS, chondroitin sulfate; PC, protein C; APC, activated protein C; EPCR, endothelial protein C receptor; RAGE, receptor for advanced glycation end-products; PAR1, protease-activated receptor 1; GPR15, G-protein coupled receptor 15; FGFR1, fibroblast growth factor receptor 1; S1PR1, sphingosine 1-phosphate receptor 1; S1P, sphingosine 1-phosphate. The figure was prepared with Biorender (premium version, Science Suite Inc-o/a BioRender, Toronto, ON, Canada).

In summary, TM can inhibit the MAPK signaling cascade in almost all cells studied either by itself or through interaction/coordination with other cell surface receptors. Promoting the generation of APC by thrombin is another key mechanism through which TM inhibits MAPK signaling and exhibits anti-inflammatory and cytoprotective effects. The activation of PAR1 by thrombin also activates MAPK pathways, which is inhibited when TM binds to thrombin. The inhibitory effects of TM on activation of MAPK signaling under the conditions of inflammation and proliferation are shown in Figure 1A. The indirect TM-mediated cytoprotective and anti-inflammatory effects, initiated by APC through activation of PAR1, are shown in Figure 1B. Noting the key roles that MAPKs play in regulation of different pathophysiological processes including proliferation, differentiation, migration survival and apoptosis, the molecules of the MAPK signaling pathways are ideal drug targets for a number of inflammatory diseases including different type of cancers. Thus, understanding the mechanisms by which TM downregulates MAPK signaling pathways may provide new strategies toward developing therapeutic drugs for cancer and other inflammatory disorders.

## Figures and Tables

**Table 1:** Role of different TM domains in regulation of MAPK signaling pathways under various conditions of inflammation and cancer.

TM (Domains)	Involved MAPKs	Condition	Used Cell Culture/Animal Model with References
Soluble full length TM (domains 1–3)	Indirect effects on p38, JNK and ERK1/2 signaling pathways	Thrombosis, inflammation and cancer	Human patients with sepsis and DIC [21,22]; histone induced mouse model of thrombosis [32]; human neutrophils and platelets (NETosis) [33]; rat model of venous thrombosis [34]; human venous thromboembolism patients [35]; human pancreatic cancer cell lines PANC-1 and MIA PaCa-2 [36]; orthotopic pancreatic cancer mouse model [36].
Lectin-like domain	p38, JNK and ERK1/2	Infection & inflammation	Allodynia mice model [27]; human embryonic kidney (HEK)-293 cells [28]; murine macrophage RAW264.7 cell line [28,29]; LPS mouse model [29]; mouse model of *Klebsiella pneumoniae* infection [29]; THP-1 cells [29]; corneal fibroblasts cells [30]; mouse model of myocardial ischemia [31]; mouse macrophages [37]; mice models of arthritis [37]; HUVECs [31,38].
EGF-like domains with serine/threonine-rich domain (domain 2 and 3)	ERK1/2	Infection & inflammation	HUVECs [39,40]; atherosclerosis mouse model [40]; LPS mouse model [41]; mouse peritoneal macrophages [41]; RAW264.7 cell line [40]; HAECs [40].
EGF-like domains 4–6 (APC generation)	p38, JNK and ERK1/2	Infection & inflammation	HUVECs [42]; porcine model of endotoxic shock [42]; rheumatoid synovial fibroblasts [43]; LPS induced Lung injury rat model [44].
C-terminal domain	p38, JNK and ERK1/2	Inflammation	THP-1 cells [45].
